# Calcium and Heart Failure: How Did We Get Here and Where Are We Going?

**DOI:** 10.3390/ijms22147392

**Published:** 2021-07-09

**Authors:** Natthaphat Siri-Angkul, Behzad Dadfar, Riya Jaleel, Jazna Naushad, Jaseela Parambathazhath, Angelia A. Doye, Lai-Hua Xie, Judith K. Gwathmey

**Affiliations:** 1 Department of Cell Biology and Molecular Medicine, Rutgers University-New Jersey Medical School, Newark, NJ 07103, USA; 2 Cardiac Electrophysiology Research and Training Center, Faculty of Medicine, Chiang Mai University, Chiang Mai 50200, Thailand; 3 Department of Physiology, Faculty of Medicine, Chiang Mai University, Chiang Mai 50200, Thailand; 4 Department of General Medicine, School of Medicine, Mazandaran University of Medical Sciences, Sari 1471655836, Iran; 5 School of International Education, Zhengzhou University, Zhengzhou 450001, China; 6 Weill Cornell Medicine Qatar, Doha P. O. Box 24144, Qatar; 7 Process Dynamics Laboratories, Al Khor 679576, Qatar; 8 Gwathmey Inc., Cambridge, MA 02138, USA; 9 Department of Medicine, Boston University School of Medicine, Boston, MA 02118, USA

**Keywords:** heart failure, excitation–contraction coupling, myocardial contractility, myofilament, sarcoplasmic reticulum Ca^2+^ ATPase, sarcoplasmic reticulum, inotrope, beta blocker, calcium transient, hypoxia

## Abstract

The occurrence and prevalence of heart failure remain high in the United States as well as globally. One person dies every 30 s from heart disease. Recognizing the importance of heart failure, clinicians and scientists have sought better therapeutic strategies and even cures for end-stage heart failure. This exploration has resulted in many failed clinical trials testing novel classes of pharmaceutical drugs and even gene therapy. As a result, along the way, there have been paradigm shifts toward and away from differing therapeutic approaches. The continued prevalence of death from heart failure, however, clearly demonstrates that the heart is not simply a pump and instead forces us to consider the complexity of simplicity in the pathophysiology of heart failure and reinforces the need to discover new therapeutic approaches.

## Introduction

**Figure 1:**
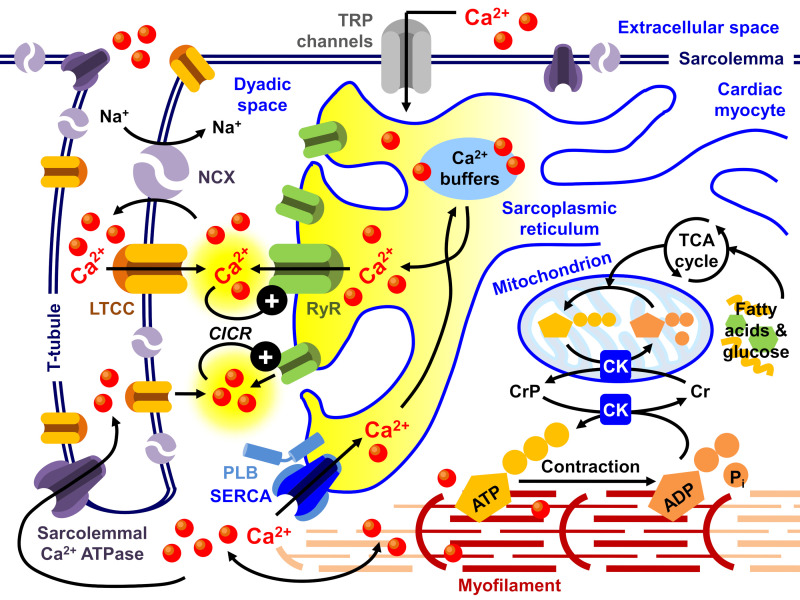
Schematic of excitation–contraction (EC) coupling in cardiac myocyte. Ca^2+^, calcium; CIRC, Ca^2+^-induced Ca^2+^ release; SERCA, sarcoplasmic/endoplasmic reticulum Ca^2+^ ATPase; PLB, phospholamban; RyR, ryanodine receptor; NCX, sodium–calcium exchanger; LTCC, L-type Ca^2+^ channel; TRP channels, transient receptor potential channels; TCA, tricarboxylic acid (Krebs) cycle; CrP, creatine phosphate; CK, creatine kinase [25,26].

Alexandre Fabiato M.D. Ph.D. was a pioneer in studying excitation–contraction (E–C) coupling in the heart and first introduced the idea of calcium (Ca^2+^)-induced Ca^2+^ release (CICR) from the sarcoplasmic reticulum (SR) [1,2,3,4,5,6,7,8,9]. According to Dr. Fabiato’s hypothesis, transsarcolemmal Ca^2+^ influx did not directly activate the myofilaments, but instead, the myofilaments were activated by a much larger amount of Ca^2+^ released from the SR. Dr. Fabiato showed that the initial relatively fast component of the transsarcolemmal Ca^2+^ current (nanomolar range) would trigger Ca^2+^ release (micromolar range) from the SR. During relaxation, the Ca^2+^ was reaccumulated into the SR by sarcoplasmic/endoplasmic reticulum Ca^2+^ATPase (SERCA), which is regulated by phospholamban as well as backed up by an efflux of Ca^2+^ across the sarcolemma through the sodium–calcium exchanger (NCX) and the sarcolemmal Ca^2+^ ATPase pump (Figure 1; also see [10,11,12,13,14]).

For many years, an ‘index’ of contractility had been sought [15,16,17,18,19]. Dr. Zia J. Penefsky demonstrated that the contraction–relaxation cycle of the heart represents the combined action of a variety of different components (Phases 1–4) in cardiac myocytes. Dr. Penefsky developed an analytical technique and showed that d*F*/d*t* (first derivative of time course of contractile force) should not be considered as the ‘index’ of contractility. She demonstrated that an increase in d*F*/d*t* can be paradoxically associated with a lower peak force, while a decrease in d*F*/d*t* can be associated with an increase in contractile response [20,21,22,23].

These initial pioneers defined key components involved in E–C coupling in the heart (Figure 1). Initially, it was thought that in patients with heart failure, the heart needed more Ca^2+^ to augment contractility. This led to the primary use of digitalis and other newly developed inotropes in conjunction with afterload reducers for the treatment of heart failure [24]. As a result, pharmaceutical companies developed positive inotropes, which were used as the main clinical approach to treating heart failure.

This review article will cover the origins of several schools of thought regarding E–C coupling in the failing heart and the treatment of heart failure [24]. We will review the role of Ca^2+^ mobilization and the abnormal contractile response seen in failing human myocardium as well as present challenges to the hypothesis that abnormal Ca^2+^ handling is solely responsible for the pathophysiology seen in failing human hearts. We will discuss how a combination of Ca^2+^ mobilization abnormalities as well as changes that reside at the level of the contractile elements (myofilaments) can interact in a dynamic manner. Furthermore, we will present how changes in myocardial energetics and changes in the intracellular milieu with regard to pH and/or phosphorylation states of key regulatory components also can contribute to the pathophysiology seen with heart failure. We will discuss how the study of animal models and human myocardium pointed to Ca^2+^ cycling abnormalities as being pivotal in the pathophysiology of human heart failure, which then resulted in a shift away from inotropes that simply increased intracellular Ca^2+^ concentration ([Ca^2+^]_i_) to the development of pharmaceutical agents that targeted the responsiveness to Ca^2+^ by the myofilaments, the use of antioxidants, and CaMKII inhibitors. We will show that despite many approaches having targeted individual/single components of E–C coupling to date, all have shown no benefit and/or differing levels of promise. This suggests a complexity to the current simplicity of thought regarding the treatment of heart failure.

## Calcium Cycling in Pressure Overload Hypertrophy, Dysthyroidism, and Human Heart Failure

Aequorin is a bioluminescent Ca^2+^ indicator that can be used in multicellular preparations [27,28,29,30,31,32,33,34]. The use of aequorin was initially applied to muscle biomedical research in the laboratory of John Rogers Blinks M.D [35,36,37]. To our knowledge, only two labs succeeded in using it successfully in animal and human myocardium (James P. Morgan M.D. Ph.D.) and vascular smooth muscle (Kathleen G. Morgan Ph.D.) [38,39]; also see [40] for a comprehensive review.

Aequorin signals were demonstrated to primarily represent SR Ca^2+^ release and re-uptake in mammalian myocardium [41,42]. Initially, phosphodiesterase (PDE) inhibitors like milrinone, amrinone, and piroximone were studied with regard to the impact on the time course of contraction and Ca^2+^ transients in papillary muscles as well as smooth muscle strips from ferrets [43,44]. Ca^2+^ transients showed an increase in peak amplitude and demonstrated an abbreviation in the time course of decline (relaxation), reflecting increased SR Ca^2+^ release and faster reuptake when stimulated by PDE inhibitors in cardiac tissue. Correspondingly, myocardial contractility demonstrated increases in peak twitch force and faster relaxation times. Hypertrophied muscles demonstrated a prolonged duration of isometric contraction and relaxation [45,46,47,48]. The increased duration of isometric contraction/relaxation in the hypertrophied muscles correlated with a similar prolongation of the Ca^2+^ transient, which was interpreted to mean that the rate of sequestration and release of Ca^2+^ by the SR was decreased in hypertrophy [46]. This study clearly demonstrated that in hypertrophied myocardium in the absence of heart failure, there was a prolongation in the Ca^2+^ transient, reflecting slowed Ca^2+^ reuptake and pointedly suggested a pivotal role for SR Ca^2+^ handling [47,48,49].

Interestingly, a study comparing weanling vs. juvenile ferrets with pressure overload hypertrophy (POH) provided insight with regard to another contributor to changes in heart function in hypertrophied myocardium [45]. Isometric twitch force, passive stiffness, [Ca^2+^]_i_, markers of myocardial energy supply, and connective tissue content were quantified. Although weanling animals with POH showed a similar degree of hypertrophy to that found in juvenile animals with POH, there were differences in peak twitch force despite there being no difference in peak [Ca^2+^]_i_. Connective tissue content, however, showed significant differences. In weanling animals with POH, connective tissue content was 10 ± 2% compared to 24.0 ± 6.8% in juvenile animals with POH (*p* < 0.001), which might have contributed to the lower peak twitch force in juvenile animals. Despite a lack of change in connective tissue content or resting [Ca^2+^]_i_ in weanling animals, passive stiffness in muscles was increased. Lactate dehydrogenase was significantly higher (38%) in weanling animals. However, total creatine kinase activity and total creatine content were significantly less (22%) in hearts from juvenile animals. This study demonstrated that despite having similar levels of hypertrophy, there can be differences in the remodeling of the extracellular matrix (increased fibrosis) as well as molecular remodeling (decrease in the creatine kinase system) [45].

In muscles from hypothyroid ferrets, peak tension was reduced and the duration of contraction prolonged [50]. These changes were associated with a decrease in amplitude and a prolonged duration of the Ca^2+^ transient. Hyperthyroidism produced opposite changes in the time course of the Ca^2+^ transient and the associated isometric contraction [50]. Gels of myosin from hypothyroid and euthyroid ferrets showed a single myosin isoform, which migrated with the slowest of the bands from the hyperthyroid ferrets (three bands). These results suggested that changes in both Ca^2+^ handling and myosin isoenzymes may contribute to contractile abnormalities and indicated a role for the contractile elements in hypertrophied myocardium [50]. These two early studies demonstrated for the first time that not only the amplitude of the Ca^2+^ transient or the amount of Ca^2+^ released from the SR, but also the time course of the Ca^2+^ transient could impact not only the time course of contraction and relaxation in the heart but the force of contraction as well [45,46,50]. Furthermore, these early studies suggested that changes at the level of the myofilaments might also contribute to differences in myocardial contractility [45,50]. Therefore, these studies suggested that the peak amplitude of Ca^2+^ released from the SR might not be the sole contributor to changes in myocardial contractility, but that changes at the level of the myofilaments might also be a contributor to observed changes in heart function.

In 1985, a cardiac transplantation program was established at the Brigham and Women’s Hospital, Harvard Medical School. After obtaining patient consent, hearts were harvested from patients undergoing heart transplantation as well as non-failing hearts from organ donors (family consent) without known cardiac disease. Wisconsin solution was used for transporting the heart samples to the laboratory. The transportation of heart samples was controlled for temperature and tissue oxygenation [51].

**Figure 2:**
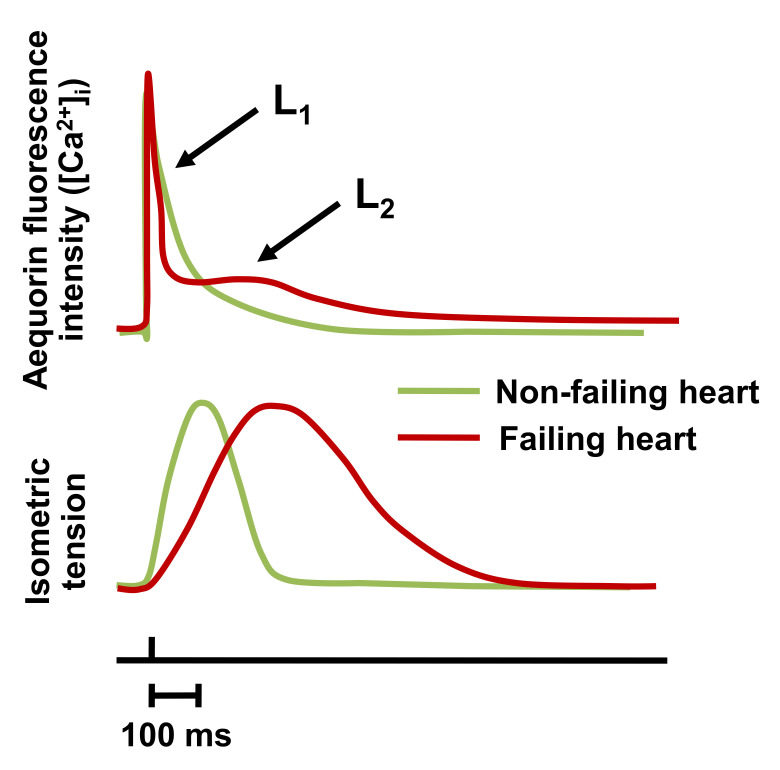
Calcium transients as detected with aequorin in trabeculae from non-failing (green) and failing (red) human myocardium with associated isometric contraction (green and red, respectively). L_1_ is the first fast component of SR Ca^2+^ release, and L_2_ is the much slower Ca^2+^ release and reuptake before restoring [Ca^2+^]_i_ to diastolic levels. Furthermore, there was a prolonged time to peak twitch force and slowed relaxation response.

For the first time, physicians and scientists observed intracellular Ca^2+^ signals in cardiac trabeculae from patients with heart failure due to ischemic and idiopathic dilated cardiomyopathy [51]. In contrast to trabeculae from non-failing human left ventricles, contractions and Ca^2+^ transients of muscles from failing hearts were markedly prolonged, and the Ca^2+^ transient exhibited two distinct components (Figure 2). Muscles from failing hearts also showed a diminished capacity to restore low resting [Ca^2+^] during diastole. These experiments provided the first direct evidence that intracellular Ca^2+^ handling is abnormal and may cause systolic and diastolic dysfunction in heart failure [49,52]. What was most striking was that, unlike earlier animal studies (pressure overload hypertrophy and dysthyroidism), the Ca^2+^ transient consisted of two components (referred to as L_1_ and L_2_) that were shown to reflect SR Ca^2+^ release and reuptake [49,51]. However, an unexpected observation was that the peak isometric contractile force was similar for the non-failing and failing human myocardium. This indicated the complexity of myocardial contractility and led to the question of whether the heart samples truly reflected global myocardial function and/or whether there were other factors involved in the systolic failure of the heart [53,54,55]. Muscle trabeculae were therefore challenged with increasing frequencies of stimulation [56,57,58,59,60]. It was demonstrated for the first time that there was indeed a negative force–interval relationship in failing human hearts that was rate dependent [56,58,60,61,62,63], and that contractile force development was a dynamic process.

Experiments were performed in human myocardium in order to investigate the relationship of intracellular Ca^2+^ handling and availability to alterations in the strength of contraction produced by changes in stimulation rate and pattern [61,62,63]. Both control and myopathic muscles exhibited potentiation of peak isometric force during the postextrasystolic contraction, which was associated with an increase in the peak intracellular Ca^2+^ transient. Frequency-related force potentiation was attenuated in cardiac muscles from patients with heart failure compared to cardiac muscles from non-failing hearts. This occurred despite an increase in resting intracellular Ca^2+^ and in the peak amplitude of the Ca^2+^ transient. Therefore, abnormalities in contractile function of myopathic muscles during frequency-related force potentiation were not due to decreased availability of intracellular Ca^2+^, but were more likely due to differences in myofibrillar Ca^2+^ responsiveness.

Sarcolemmal Ca^2+^ influx might have also contributed to frequency-related changes in contractile force in myopathic muscles as suggested by a decrease in action potential duration with increasing stimulation frequency, which was associated with fluctuations in the peak Ca^2+^ transient amplitude [58,64]. Furthermore, it was found that a complex exponential function best fit Ca^2+^ transients at higher frequencies of simulation. It was suggested that NCX was not the source of the Ca^2+^ and the possibility was raised that restitution of the contractile response was related to Ca^2+^ supplied from some other source. This suggested that not only the Ca^2+^ released from SR and Ca^2+^ mobilized by the NCX, but Ca^2+^ from another source might be involved. These Ca^2+^ channels may later have been identified to be transient receptor potential canonical (TRPC) channels [65,66,67,68,69,70,71,72], which have been reported to mediate store-operated Ca^2+^ entry (SOCE) and mechano-electrical feedback in the cardiomyocyte [73,74,75,76,77,78,79,80]. Moreover, the mechanistic link between TRPCs and Ca^2+^ mishandling has been reported in multiple experimental models of cardiac disease including myocardial infarction [66,81,82,83,84,85,86,87], atrial fibrillation [88,89,90,91], and iron overload cardiomyopathy [92]. In addition to TRPCs, other members of the TRP superfamily [93,94,95], e.g., melastatin (TRPM) [96,97,98,99,100,101,102,103,104] and vanilloid (TRPV) subtypes [105,106,107,108,109,110,111,112,113,114,115,116], are also potential candidates mediating the non-canonical Ca^2+^ entry into cardiomyocytes.

There remains controversy as to whether the regulation of SERCA activity is altered in human myocardium from patients with heart failure or whether decreased SERCA activity is due to changes in SERCA or phospholamban expression. Cyclic adenosine monophosphate (cAMP)-dependent phosphorylation of phospholamban has been proposed to be in part responsible for the reduced SERCA activity in human heart failure [117,118]. It was reported that the protein levels of phospholamban and SERCA were unchanged in failing compared with non-failing human myocardium. A decrease in responsiveness to the direct activation of SERCA activity by either cAMP or protein kinase A (PKA) was also reported in failing myocardium. From these observations, it was concluded that the impaired SR function in human heart failure might be due to reduced cAMP-dependent phosphorylation of phospholamban resulting in a difference in SERCA activity [117,118].

Two of the most significant characteristics of failing human myocardium that have been reported are an increase in diastolic [Ca^2+^] and a prolonged diastolic relaxation. These abnormalities are more pronounced at higher frequencies of stimulation and may be caused by an altered Ca^2+^ resequestration into the SR. SERCA activity has been reported to be positively correlated with diastolic force. Diastolic force has an inverse relationship with the SERCA activity in failing myocardium. These findings suggested that a reduction in SERCA activity could contribute to both an impairment in systolic as well as diastolic function in failing human hearts [117].

Connecting the [Ca^2+^]_i_ availability and myofilament contractile activation is cross-bridge cycling dynamics (Figure 1). In skinned muscle fibers, a maximal Ca^2+^-activated force was found to be similar between muscle fibers from non-failing and failing human hearts. Dynamic stiffness was larger in muscles from failing human hearts. Cross-bridge cycling rates were significantly slower in muscles from failing hearts compared to muscles from non-failing human hearts [119]. It was suggested that because failing hearts have a markedly diminished energy reserve, the slowing of the cross-bridge cycling rate might reflect changes in contractile protein composition. Yet again, the potential to generate maximal Ca^2+^ activated force was found to be similar in non-failing and failing human muscle strips, raising once more the question of whether contractility was depressed in the failing human heart [120,121].

**Figure 3:**
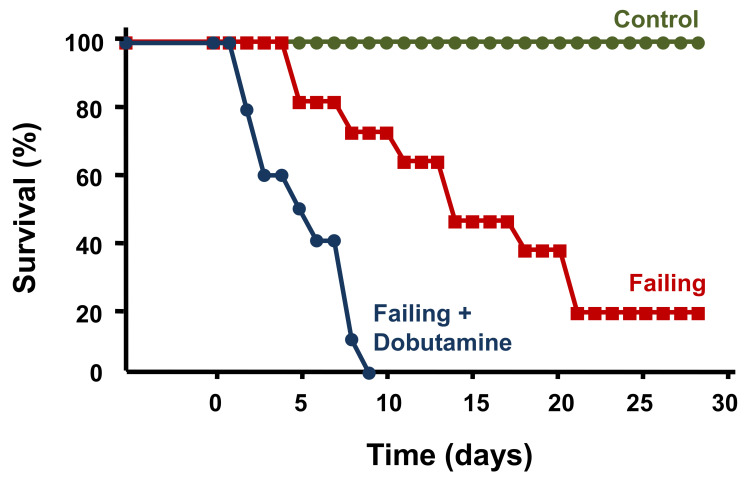
Dobutamine effect on survival. Heart failure was induced in rats by banding the aorta. Dobutamine is a catecholamine that acts on α_1_, β_1_, and β_2_ adrenergic receptors. Stimulation of these receptors by dobutamine produces a relatively strong inotropic effect. Treatment by dobutamine resulted in a significantly higher and earlier mortality rate. This demonstrates the negative impact of increased inotropy in the treatment of heart failure.

The question was then asked whether the pharmacological agent milrinone (a PDE inhibitor) might be beneficial in the treatment of heart failure. Studies on isolated muscles from failing human hearts demonstrated a decrease in inotropic response to milrinone [122]. In order to attain a beneficial effect, extremely high concentrations were needed as well as an increase in cAMP. Adenylate cyclase was therefore stimulated using forskolin. Only then did an increase in cAMP restore myocardial contractility in muscles from failing human hearts and responsiveness to milrinone. It was predicted based on studies using muscle preparations from failing human hearts that the PROMISE trials using milrinone would likely fail and show toxicity, as it was designed to include digitalis, which it did [122,123]. Our prediction of toxicity and reduced inotropic response was borne out in clinical trials despite the considerable fanfare afforded to milrinone at the American Heart Association for the use of PDE inhibitors as a new class of agents to be used as clinical tools to treat heart failure [123,124]. To the clinical community and pharmaceutical industry, this failure was a severe and disheartening disappointment [123]. Despite milrinone’s beneficial effects on hemodynamics, long-term therapy with milrinone was shown to increase morbidity and mortality in patients with severe chronic heart failure. The class of drugs referred to as PDE inhibitors appeared to exert deleterious effects and is currently used primarily as a bridge to cardiac transplantation. Animal studies also showed that dobutamine, which is often used to treat acute heart failure, resulted in increased mortality (Figure 3) [125]. Dobutamine is known to produce a relatively strong inotropic effect via stimulating primarily β_1_ and β_2_ adrenergic receptors. Its clinical use is often referred to as dobutamine holidays [126].

## Inotropes vs. Beta Blockers in the Treatment of Heart Failure: Paradigm Shift

**Figure 4:**
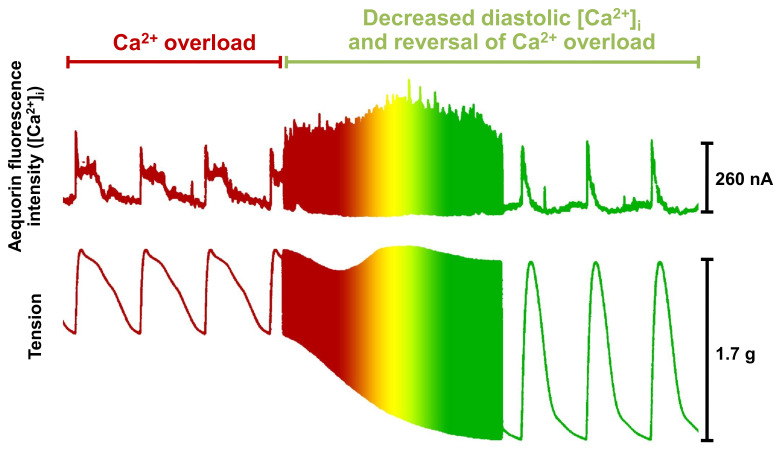
The impact of calcium overload on systolic and diastolic function. In trabeculae from a patient with heart failure, there was a slowed contractile response as well as slowed reuptake of Ca^2+^ by the SR and delayed return to normal diastolic Ca^2+^ levels. When diastolic Ca^2+^ concentration was reduced and restored to pre-Ca^2+^ loading concentrations, there was first an overshoot in the Ca^2+^ transient amplitude showing increased SR Ca^2+^ re-uptake, which was followed by a normalization of amplitude and time course of the Ca^2+^ transient. Associated with these changes in Ca^2+^ transient amplitude and time course and a decrease in diastolic Ca^2+^ concentration, there was a tremendous increase in contractile force. This figure demonstrates the negative impact of elevated diastolic Ca^2+^ on not only diastolic force and muscle relaxation, but systolic force as well.

Data were pointing to a potentially detrimental effect from increasing [Ca^2+^]_i_ and enhancing inotropy/myocardial contractility in heart failure patients. A simple increase in intracellular Ca^2+^ was being shown to be clinically detrimental (Figure 4). The clinical and scientific communities were beginning to understand that abnormal Ca^2+^ handling was more complex. Agents targeting the myofilaments referred to as Ca^2+^ sensitizing agents showed little beneficial effect and actually pointed to possible detrimental outcomes [53,127,128,129,130,131,132]. After careful review of animal models of cardiomyopathy, two avian models of heart failure were selected for comparative studies addressing controversial findings reported in myocardium from failing human hearts [133,134,135]. One was induced by furazolidone (700 ppm) added to the food, and the other occurred spontaneously in turkey poults (resembling idiopathic dilated cardiomyopathy) [136]. Furazolidone-induced cardiomyopathy and spontaneous dilated cardiomyopathy in turkey poults shared significant similarities to human heart failure at the cellular, subcellular, receptor, and organ levels [135,136,137,138,139,140,141,142,143,144,145,146,147,148,149,150,151].

β-Adrenergic blocking agents had been approved for clinical use in the treatment of hypertension [152,153,154,155,156], coronary artery disease [157,158,159,160,161], and arrhythmias [162,163,164]. However, the underlying mechanisms of potential beneficial effects of β-blockers in heart failure remained unsolved [165]. The use of β-blockers in animal models of heart failure had been investigated, but the results were found to be ambiguous. For example, in the Syrian hamster model of cardiomyopathy, β-blocker treatment showed no benefit in preventing the development of heart failure [166]. However, early studies showed that β-blockade with propranolol was cardioprotective and prevented the development of dilated cardiomyopathy (DCM) in turkey poults [137,141]. The avian models similar to human idiopathic DCM showed left ventricular dilatation, decreased ejection fraction, left ventricular free wall thinning, decreased β_1_-receptor density, decreased SERCA, decreased myofibrillar ATPase activity, and reduced metabolism markers [142]. Histologically, there was myocyte hypertrophy and increased interstitial fibrosis. The animals also showed, unlike many animal models of heart failure, cachexia. Furazolidone was later shown to impair SERCA activity [167].

**Figure 5:**
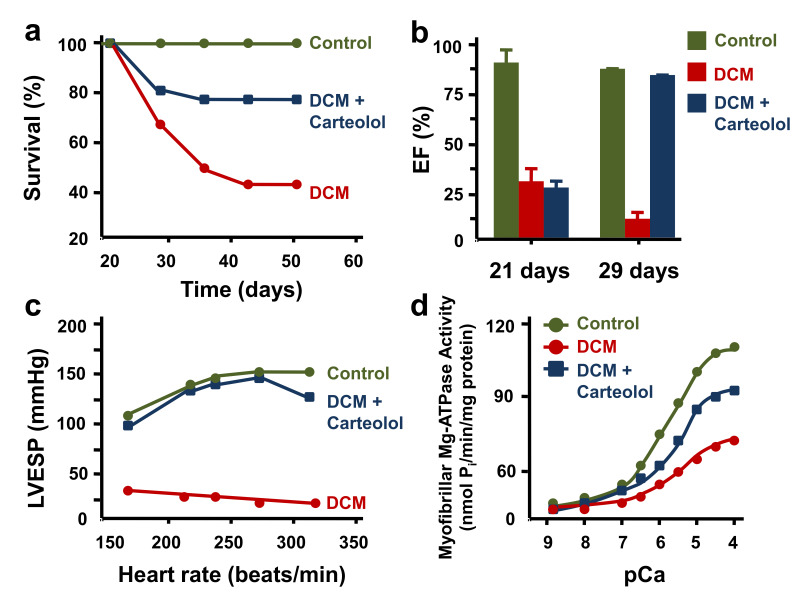
Treatment of animals with dilated cardiomyopathy with carteolol, a beta blocker. (**a**) Survival of turkey poults with furazolidone-induced heart failure (DCM). Carteolol is a β-blocker. Animals treated with carteolol had significantly improved survival (blue) compared to untreated animals with heart failure (red). (**b**) Effect of carteolol on echocardiography-derived ejection fraction. Animals with heart failure treated with carteolol had restored ejection fractions (blue) compared to untreated animals with heart failure (red). Untreated animals continued to have worsening of their heart failure, demonstrating that there was progressive heart failure (red, 29 days). (**c**) Left ventricular end-systolic pressure was also maintained in turkeys treated with carteolol (blue) compared to non-treated animals with heart failure (red). (**d**) Changes at the level of the contractile elements may explain in part the beneficial effects seen with carteolol treatment. Myofibrillar Ca^2+^ ATPase was significantly reduced in animals with heart failure (red). Carteolol treatment tended to restore myofibillar Ca^2+^ ATPase activity (blue) to levels similar to what was seen in control animals (green). Furthermore, β-adrenergic receptor density, forced interval relationships, SERCA activity, and myocardial energetics were restored with carteolol treatment.

Carteolol, a β-adrenergic blocking agent was tested in turkey poults with DCM (Figure 5). There was 59% mortality in the untreated DCM group and 22% mortality in the group treated with carteolol. The treated group showed a restoration of ejection fraction and left ventricular peak systolic pressure [142]. Carteolol treatment increased β-adrenergic receptor density, decreased the amount of connective tissue (fibrosis), restored SERCA and myofibrillar ATPase activities, and restored creatine kinase, lactate dehydrogenase, aspartate transaminase, and ATP synthase activities. It is important to note that a lower dose of carteolol did not restore heart function despite restoring β-receptor density, energy metabolism, and SERCA and ryanodine receptor (RyR) protein expression. These data suggest that adaptations in myocardial energy metabolism and Ca^2+^ cycling proteins are more quickly restored than the negative impact at the level of the contractile elements (i.e., myofibrillar ATPase activity), which may explain in part the failure of the gene therapy trial (CUPID) using SERCA2a in heart failure patients [168,169,170,171]. The duration and extent of continued expression of SERCA2A may have been reduced over time despite early Phase 1 studies having shown continued expression in cardiac biopsies. Changes at both the level of the thick and thin myofilaments appear to be important in the pathophysiology of heart failure and must be addressed if there is to be the development and successful clinical testing of new therapeutic agents [53]. This study was the first to show that β-blockade could improve survival, reverse contractile abnormalities, and induce cellular remodeling. Furthermore, it showed that end-stage heart failure could be reversed.

This observation began the paradigm shift towards the use of β-blockers for the treatment of heart failure. Ca^2+^ sensitizers (e.g., MCI-154 and DPI201-106) as well as PDE inhibitors (e.g., amrinone, milrinone, and piroximone) had been shown to have potential detrimental effects in failing human myocardium [53,127,129,131,132,172,173]. The paradigm shift resulted in a number of clinical studies that showed that β-blockers had long-term beneficial effects in patients with heart failure. In a multicenter trial, the β_1_-selective antagonist metoprolol was found to reduce the combined end point of death and the need for transplantation in patients with idiopathic DCM [174]. Another large multicenter clinical trial, in which the nonselective β-blocker carvedilol was used in patients with heart failure, showed a 65% reduction in mortality [175].

## Myofilaments and Ca^2+^ Responsiveness

It was the labs of Leslie Leinwand and Michael Bristow that first identified changes in myosin isoforms in myocardium from patients with heart failure. They found that the relative amounts of the α isoform of myosin heavy chain (α-MyHC) were significantly lower or non-existent in failing hearts regardless of the cause of heart failure [176]. They posited that this molecular alteration may be sufficient to explain systolic dysfunction in failing hearts and that therapeutics targeting increasing α-MyHC gene expression might be a feasible therapeutic approach [176]. This landmark work by Leinwand and Bristow set the stage for consideration that changes at the level of the contractile elements might contribute in a significant way to the pathophysiology of human heart failure and that [Ca^2+^]_i_ and SR Ca^2+^ release were not the sole contributors to heart failure. A novel mutation in TnC was also reported that further strengthened this school of thought [177]. To address the potential functional changes in myocardial contractility as a result of reported changes that resided at the level of the myofilaments, both skinned as well as intact myocardium from failing human hearts were studied [178]. Ca^2+^ concentration required for 50% activation and Hill coefficient for fibers from non-failing and failing human hearts at pH 7.1 were not different. Maximum Ca^2+^-activated force again was not different. However, at lower intracellular pH (6.8 and 6.9), differences were seen in myofilament Ca^2+^ activation between non-failing and failing hearts. At lower intracellular pHs, failing myocardium was shifted left on the Ca^2+^ axis, indicating an increase in myofilament Ca^2+^ responsiveness. Increases in inorganic phosphate, cAMP, and PKA stimulation impacted Ca^2+^ responsiveness differently in non-failing vs. failing human myocardium. Interestingly, the combination of PKA stimulation in the presence of increased [cAMP] resulted in a further rightward shift in non-failing human myocardium, but did not further shift the Ca^2+^-force relationship in fibers from failing hearts. Importantly, cyclic guanosine monophosphate (cGMP) resulted in a greater decrease in myofilament sensitivity in fibers from failing hearts. From these studies, it was suggested that not only changes at the level of the thick myofilaments, as previously reported by Leinwand and Bristow [176], but also changes at the level of the thin myofilaments could result in differential responses to changes in the intracellular Ca^2+^milieu in failing myocardium. Furthermore, changes at the level of the myofilaments might also contribute to the reduced cross-bridge cycling rate that had been reported earlier in failing human myocardium [119].

Studies on Mg-ATPase and Ca^2+^-activated myosin ATPase activity in failing human myocardium and the effect of agents referred to as Ca^2+^ sensitizers provided insights into the impact on the contractile elements in patients with heart failure and were confirmed in an animal model reflecting the pathophysiology and subcellular remodeling being discovered in failing human hearts [53,150,173]. These studies revealed differences between disease states (idiopathic vs. ischemic cardiomyopathy) as well as suggested that the thin myofilament component TnI might be important. It was proposed that myosin light-chain-related regulation might also play a complementary role in the troponin-related regulation of myocardial contractility [53,150].

Protein kinase C (PKC) activation is known to be a major regulator of vascular smooth muscle function [39,179]. Furthermore, activated PKC phosphorylates different substrates including ion channels and pumps. PKC has yet to be targeted for the treatment of heart failure. PKC is known to phosphorylate both troponin I (TnI) and troponin T (TnT). In human myocardium, PKC activation was shown to result in a decrease in myofilament Ca^2+^ sensitivity as well as a decrease in maximal Ca^2+^-activated force [180]. Stimulation of PKC also resulted in a decrease in peak isometric twitch force and peak SR Ca^2+^ release. Further, in the presence of 12-deoxyphorbol 13 isobutyrate 20 acetate (DPBA), a PKC stimulator, the steady-state force-Ca^2+^ relationship was shifted to higher [Ca^2+^]_i_, and there was a change in the Hill coefficient (myofilament cooperativity). These findings indicated that there was a decrease in myofilament Ca^2+^ sensitivity and a change in cooperativity among thin myofilament proteins most likely reflecting phosphorylation of thin filament regulatory proteins by PKC. Both TnT and TnI have at least two phosphorylation sites. In heart failure tissue, there are two TnT isoforms. These findings might reflect the effect on relative states of phosphorylation of two TnT isoforms. PKC activation, which decreased SR Ca^2+^ release while decreasing the Ca^2+^ responsiveness of the myofilaments with a change in cooperativity among thin myofilament proteins, might be a future approach to the treatment of heart failure and Ca^2+^ regulation in the failing heart. However, the impact of PKC stimulation on vascular smooth muscle remains unclear and must be considered before possible future clinical development [39,40,179]. Some reports suggest vasodilation while others suggest a vasoconstrictive effect with PKC stimulation [181].

Over twenty years ago, as early as 2000, the effect of cGMP on failing human myocardium was reported [180,182,183]. cGMP stimulates cAMP-dependent PDE, which results in an increase in cAMP hydrolysis [183]. cGMP activation of cGMP-protein kinase results in the phosphorylation of TnI and a decrease in myofilament Ca^2+^ responsiveness. As failing myocardium had been reported to have significantly less cAMP [122], a lesser inhibitory effect would be expected for the cGMP-induced decrease in Ca^2+^ sensitivity. Potent drugs are now available to augment these signaling systems with conventional soluble guanylate cyclase (sGC) stimulators, novel NO-independent sGC stimulators, GC-A and GC-B agonists, and PDE inhibitors [182,184,185,186]. Guanylate cyclase has recently become a new target for the treatment of heart failure [187,188]. In clinical trials, the incidence of death from heart failure has been reported to be lower for patients receiving vericiguat, a guanylate cyclase stimulator [188]. Although the mechanism of action of its beneficial effects in heart failure is still reported as being unknown, with vascular effects being highlighted, it has been reported that increasing cyclic GMP in skinned fibers from failing human hearts resulted in a decrease in myofilament Ca^2+^ sensitivity with no change in maximal Ca^2+^ activated force [178].

Despite indications that changes at the level of the thick and thin myofilaments and diastolic [Ca^2+^] and SR Ca^2+^ release are key contributors to the pathophysiology of heart failure and possible targets for therapeutic intervention, there is a dynamic state that occurs in the failing heart, especially when blood circulation is impaired and when increases in heart rate can induce a hypoxic milieu [189,190,191]. Changes in intracellular pH, phosphate levels, and phosphorylation states in the heart are dynamic processes all of which must be taken into consideration when treating heart failure. Myocardial energetics changes in a dynamic way both on the demand and the utilization side of the equation (Figure 1) in response to changes in heart rate and other pathophysiological stresses [25,26,59,137,148,192,193,194]. It is the complexity of simplicity in thinking applied to understanding heart failure and regarding the heart only as a pump that needs to be addressed if safe and efficacious therapeutics are to be attained. Herd thinking will not get us there. Although the phenotype of heart failure is an enlarged heart with poor contractility, the molecular phenotype and response to functional stresses must be studied. Animal models reflective of the human condition must be used in pre-clinical development and testing of new therapies. It is because of possible detrimental impacts on the heart and possible fatal consequences that every drug during pre-clinical development must be tested for cardiotoxicity [147,195]. Cardiotoxicity remains a major decision factor for success or failure in clinical drug development and successful marketing (e.g., in the case of the COX-2 inhibitor rofecoxib for arthritis [196,197,198,199,200]).

## Antioxidants and CaMKII Inhibitors in the Treatment of Heart Disease

Calcium/calmodulin-dependent protein kinase II (CaMKII) is a serine/threonine kinase that has emerged as a key regulator of cardiac physiology and pathology [201,202,203,204]. CaMKII can be activated by phosphorylation, oxidation, or *O*-linked *N*-acetylglucosamine modification [205,206]. Excessive activation of CaMKII has been suggested to promote the development of cardiac diseases including cardiac hypertrophy, heart failure, and cardiac arrhythmias [207,208,209,210,211]. Activation of CaMKII has been shown to promote late Na current, late-phase L-type Ca^2+^ current, and RyR Ca^2+^ leak, which facilitate the progression of the above-mentioned diseases [207,212]. Therefore, CaMKII has been considered as a target for the treatment of arrhythmias and heart failure [213,214]. Beneficial effects of CaMKII inhibition have been observed in experiments using animal models and cardiac myocytes from heart failure patients [214,215,216,217]. However, a recent report on the first clinical trial (Phase II) with the CaMKII inhibitor (NP202) revealed no prevention of post-MI heart failure [218]. One reason might be that NP202 did not substantially inhibit CaMKII due to its low potency [219]. Future clinical studies using high-potency CaMKII inhibitors or genetic strategies are warranted to reveal the potential of CaMKII inhibitors as a therapeutic approach to cardiovascular diseases.

A main upstream regulator of CaMKII is the oxidation at M281/282 by reactive oxygen species (ROS) [205,220,221]. While ROS are necessary for normal cellular functions, excessive levels of ROS induce oxidative stress and cause damage to DNA, lipids, and proteins, and thus promote cardiovascular diseases [222]. Therefore, focusing on oxidative stress (e.g., use of antioxidants) remains a potential therapeutic strategy in treating cardiovascular diseases [223,224,225].

## Conclusions

Despite progress in the clinical management of heart failure, it continues to be associated with a high rate of mortality and morbidity worldwide [226,227,228]. Much remains to be understood regarding heart failure and its treatment by targeting receptors (e.g., angiotensin II receptor blockers (ARBs), angiotensin-converting enzyme inhibitors (ACEIs), angiotensin receptor-neprilysin inhibitors (ARNIs), β-adrenergic receptor blockers, and α-adrenergic receptor blockers), guanylate cyclase stimulators, and CaMKIIδ inhibitors [218,219]. Combination therapies still leave much to be desired. Positive inotropic agents still remain major players in the clinical management of heart failure, especially in patients with acute decompensation [229,230,231,232]. A recent clinical trial “CUPID” used gene therapy to target the major Ca^2+^ cycling protein SERCA2a [233,234,235], yet failed in Phase IIb trials despite having shown promise in early Phase 1 trials [168,169,170,171,236,237,238,239,240]. The clinical and research communities need to better appreciate the complexity of a simplicity in thinking when it comes to heart failure and future perspectives for the development of targeted gene therapy or other therapies for its treatment and/or prevention. Will end-stage heart failure forever remain or need to remain end-stage?

## Data Availability

Not applicable.
